# Community-based interventions to increase dairy intake in healthy populations: a systematic review

**DOI:** 10.1186/s40985-020-00135-4

**Published:** 2020-08-04

**Authors:** Zeinab Nikniaz, Jafar Sadegh Tabrizi, Morteza Ghojazadeh, Mahdieh Abbasalizad Farhangi, Mohammad-Salar Hosseini, Motahareh Allameh, Soheila Norouzi, Leila Nikniaz

**Affiliations:** 1 Liver and Gastrointestinal Diseases Research Center, Tabriz University of Medical Sciences, Tabriz, Iran; 2 Tabriz Health Services Management Research Center, Faculty of Management and Medical Informatics, Tabriz University of Medical Sciences, Tabriz, Iran; 3 Research Center for Evidence-Based Medicine, Tabriz University of Medical Sciences, Tabriz, Iran; 4 Drug Applied Research Center, Tabriz University of Medical Sciences, Tabriz, Iran; 5 Student Research Committee, Tabriz University of Medical Sciences, Tabriz, Iran; 6 Adolescent, Youth and Schools Health Office, Ministry of Health and Medical Education, Tehran, Iran; 7 Department of Human Nutrition, Faculty of Medicine, Qazvin University of Medical Sciences, Qazvin, Iran

**Keywords:** Community-based interventions, Dairy, Milk, Systematic review

## Abstract

**Background:**

Considering the low frequency of dairy intake in the population, interventions aiming to increase its consumption can be a priority for any health system.

**Objective:**

This study aims to summarize community-based interventions for improving dairy consumption and their effectiveness to help policy-makers in designing coherent public health strategies.

**Methods:**

This study was conducted in 2019, using PubMed/MEDLINE, Scopus, EMBASE, Cochrane Library, Web of Science, ProQuest, and Google Scholar. Two independent reviewers selected the eligible studies, and the outcomes of interest were extracted. The quality of eligible studies was assessed using the Joanna Briggs Institute Critical Appraisal Checklist for randomized controlled trials and quasi-experimental studies.

**Results:**

Out of 521 initially identified articles, 25 studies were included. Interventions reported in 19 studies were effective in increasing dairy consumption. Interventions in high-income countries were more effective than those in middle- and low-income countries. Interventions in health centers and supermarkets were more effective than the community and school-level interventions. Interventions in supermarkets and adolescents as target groups were more effective than children, middle-aged people, and the elderly. Also, educational interventions and changing buying/selling pattern were more effective than multiple interventions. Interventions longer than 24 and 48 weeks were more effective than shorter interventions.

**Conclusion:**

Three policy options including educational interventions, multiple interventions, and changing the purchase pattern are suggested. It seems that applying all of the interventions together can be more effective. Also, long-term and well-designed future studies in different settings are recommended to confirm these results.

## Background

Dairy is a rich source of proteins, vitamins, and minerals. It fulfills the vital needs of humans in different periods of life. So, consuming dairies has a great importance regardless of age [1]. Milk and dairy products are rich sources of calcium and magnesium [2]. These minerals are essential for the growth and strength of the bones, especially in children and adolescents due to rapid skeleton growth and bone condensation [3]. In addition, calcium is necessary for the natural mineralization of the bones and the matrix of cartilage [4]. According to the evidence, calcium can also reduce the risk of osteoporosis in the older population [5–8]. In adulthood, consuming dairy is associated with the risk reduction for some chronic diseases [9, 10]. Increased intake of dairy products using a restricted diet can accelerate weight loss [11, 12]. The results of a systematic review showed that an average increase in the consumption of low-fat dairy (200 g per day) reduces the risk of diabetes [13]. Also, a meta-analysis of cohort studies demonstrated that total milk and dairy consumption have an inverse relation with the incidence of colorectal cancer [14]. Since dairy products have a proper amount of calcium, magnesium, and potassium—which play an important role in regulating blood pressure—they can be useful in decreasing blood pressure and preventing stroke [15].

According to studies, dairy intake is considered low among children and adolescents [16, 17]. According to the United States Department of Agriculture (USDA), the daily recommendation of dairy for an adult with an energetic need of 2000 Kcal/day is 3 portions per day, but the US population only consumes half of the recommendation (1.5 portions per day) [18]. Also, some other studies conducted in different age groups indicated that the intake was below the recommended daily amount of dairy [19–28].

Although dairy consumption has countless benefits for improving health issues, the amount of dairy intake is lower than the international recommendations [22–24, 29], which can lead to an increase in the incidence of certain diseases such as osteoporosis, diabetes, colorectal cancer, and hypertension as well as healthcare costs [30]. In order to identify policy options to increase dairy intake, reviewing successful community-based strategies and their effectiveness is needed to help policy-makers in choosing the best policy options with respect to situations in each country. Considering the numerous evidence available and the lack of a systematic review in this field, this study was conducted to evaluate community-based interventions for improving dairy consumption and their effectiveness to help policy-makers in designing coherent public health strategies.

## Methods

The present systematic review attempted to evaluate the effect of community-based interventions to increase dairy intake in all population groups. The main outcome of the present study was an increase in the amount of dairy intake in a healthy population. The reporting procedures were according to the guidelines presented by Preferred Reporting Items for Systematic Reviews and Meta-Analysis (PRISMA) (Supplementary material) [31].

### Search strategy

This systematic review was designed and conducted in 2019. Such databases as PubMed, Scopus, Google Scholar, Cochrane Library, Science Direct, and Web of Science were systematically searched. The timeframe selected for searching articles was from 2000 to 2019. A number of prestigious journals in the field were also searched manually to identify and cover more articles, mainly the ones published after the database search was performed. After excluding articles which did not meet the inclusion criteria, the reference lists of the remaining articles were also searched to increase the reliability of identifying and reviewing the eligible articles. The databases of the European Association for Gray Literature Exploitation (EAGLE) and the Health Care Management Information Consortium (HMIC) were also searched for gray literature. The search strategy for PubMed was as follows: Search (dairy[Title/Abstract]) OR yoghurt[Title/Abstract]) OR yogurt[Title/Abstract]) OR milk[Title/Abstract] OR calcium[Title/Abstract]) AND "community based"[Title/Abstract]) OR community-based[Title/Abstract]) OR population-based[Title/Abstract]) OR evidence-based[Title/Abstract]) OR "population based"[Title/Abstract]) OR practice[Title/Abstract]) OR education[Title/Abstract]) OR "food policy"[Title/Abstract]) OR intervention[Title/Abstract]) OR implementation[Title/Abstract]) OR approach[Title/Abstract]) OR strategy[Title/Abstract]) AND "national program"[Title/Abstract]) OR country[Title/Abstract]) OR community[Title/Abstract]) NOT sheep[Title/Abstract]) NOT farm[Title/Abstract]) NOT cow[Title/Abstract]) NOT herd[Title/Abstract]) NOT lactation[Title/Abstract]) NOT newborn[Title/Abstract]) NOT infant[Title/Abstract]

### Inclusion and exclusion criteria

Randomized clinical trials (RCTs) or quasi-experimental studies were included if they (i) enrolled all age groups of normal and healthy population, (ii) evaluated the effect of community-based intervention to increase dairy consumption, and (iii) were published in an English language journal.

Studies conducted on subjects with chronic diseases such as cardiovascular diseases, diabetes, and hypertension were excluded from the study. Also, studies which focused on calcium intake instead of dairy consumption were removed. Economic evaluations, modeling studies, laboratory and observational studies, and studies with the aim of tool development were also excluded. The research question based on PICO is available in Table 1.

**Table 1:** Inclusion and exclusion criteria based on PICO

PICO components	Inclusion criteria	Exclusion criteria
**Population**	All ages and genders	Patients referring to healthcare centers
**Intervention**	Community-based interventions in order to increase dairy/calcium consumption	Clinical or laboratory interventions, intervention on reducing the consumption of high-fat dairy
**Comparison**	All ages and genders	Patients referring to healthcare centers
**Outcome**	Increase in amount or times of dairy consumption	Reducing the consumption of high-fat dairy, increasing awareness, etc.
**Other criteria**	Publications in English	Observational studies, studies with the aim of tool development

### Study selection, data extraction strategy, and quality assessment

**Fig. 1:**
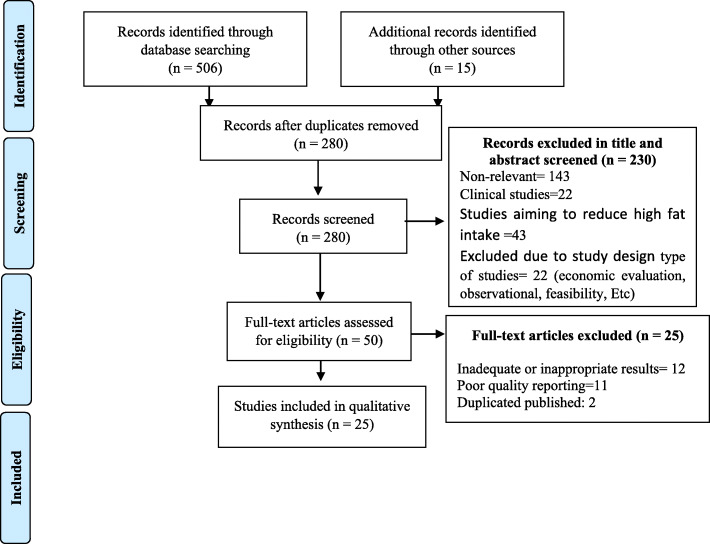
Searches and inclusion process flow diagram

Two reviewers extracted the data independently and screened the title and abstract of records to identify which potentially relevant records met the inclusion/exclusion criteria. Full-text articles were obtained for these records and were independently assessed for relevance by the reviewers. The following information was extracted from the studies: author(s), year of publication, target population characteristics, country, methodological characteristics (study design), types and aims of intervention, and main outcomes. Countries were categorized as low-, middle-, and high-income according to the World Bank [32] for the current 2019 fiscal year. “Low-income economies were defined as those with a gross national income (GNI) of $995 or less in 2017, which was calculated using the World Bank Atlas method; lower middle-income economies were those with a GNI per capita between $996 and $3895; upper middle-income economies were those with a GNI per capita between $3896 and $12,055; and high-income economies were those with a GNI per capita of $12,056 or more”. The quality of studies was assessed using the Joanna Briggs Institute Critical Appraisal Checklist for RCTs and Quasi-Experimental Studies. A flow chart of the literature review is shown in Fig. 1.

## Results

Out of 521 results retrieved from databases and other mentioned sources, 241 were removed due to the duplicity among different sources. Reviewing the title and abstract of the results, 230 more studies were excluded due to incompatibility with the study question and aim. After reviewing the full texts of the remaining articles, 25 other studies were removed; finally, 25 articles were included in the study (Fig. 1).

The data and characteristics of the 25 reviewed articles are presented in Tables 2 and 3. Out of the 25 reviewed articles, 23 were in high-income countries. Most studies (*n*: 16) were conducted in the USA. The studies could be divided into four categories based on the context: at the community level (*n*: 6), schools (*n*: 11), health centers (*n*: 5), and markets (*n*: 3). Also, most studies (*n*: 16) were randomized controlled trials.

**Table 2:** Characteristics of the included studies

Author, year	Country	Setting	Study design	Participants	
				Intervention	Control
**Kimura, et al. 2013[**33**]**	Japan	Community	RCT	Older adults aged 65–90 years	
				57	35
**Duncanson, et al. 2013[**34**]**	Australia	Health car facilities	RCT	Parent-child dyads aged 2.0–5.9 years)	
				75	71
**McCarthy, et al. 2007[**35**]**	USA	Community	RCT	Predominantly overweight or obese, healthy women aged 23–77 years	
				188	178
**Casazza, et al. 2006[**36**]**	USA	High schools	Qu	Adolescent students aged 14–19 years	
				2565	1599
**DeBar, et al. 2006[**37**]**	USA	Health care facilities	RCT	Students aged 14 to 16 years	
				101	108
**DeBar, et al. 2009[**38**]**	USA	Health care facilities	RCT	Students aged 14 to 16 years	
				82	41
**Dawson 2006[**39**]**	USA	Middle school	Qu	Students in grades 7 and 8	
				45	18
**Gates, et al. (1) 2013[**40**]**	Canada	Middle school	Qu	Students aged 11–15 years	
				26	No control
**Gates, et al. (2) 2013[**41**]**	Canada	Middle school	Qu	Students in grades 6 to 8	
				70	No control
**Lo, et al. (2008)[**42**]**	Canada	Middle school	Qu	Students in grade 9	
				53	48
**Naghashpour, et al. 2014[**43**]**	Iran	High school	RCT	Female students in junior high school	
				95	93
**Olson, et al. 2008[**44**]**	USA	Health care facilities	RCT	Healthy teens aged 11–20	
				136	148
**O’Connell, 2005[**45**]**	USA	Middle school	RCT	Students in grade 7	
				220	269
**Singhal, et al. 2010[**46**]**	India	High school	RCT	students in grade 11	
				99	102
**Watson, et al. 2009[**47**]**	USA	High school	Qu	Students aged 14–19 years	
				45	30
**Wordell, et al. 2012[**48**]**	USA	Middle school	Qu	Students in grades 7 and 8	
				1406	2707
**Yamaoka, et al. 2011[**49**]**	Japan	Community	RCT	Female adolescents aged 13–15	
				225	234
**Finnell, et al. 2017[**50**]**	USA	Supermarkets	Qu	Supermarkets	
				80	66
**Bernstein, et al. 2002[**51**]**	USA	Community	RCT	Men and women older than age 69	
				38	32
**Ni Mhurchu, et al. 2010[**52**]**	New Zealand	Supermarkets	RCT	Shoppers (adults)	
				277	278
**Foster, et al. 2014[**53**]**	USA	Supermarkets	RCT	Supermarkets	
				4	4
**Freedman and Nickell. 2010[**54**]**	USA	Community	Qu	children aged 9 to 14 years	
				49	–
**Friedman, et al. 2007[**55**]**	USA	Health care facilities	RCT	Children aged 8–10 years	
				237	249
**Hovell, et al. 2009[**56**]**	USA	Community	RCT	Children aged 10–13 years	
				68	49
**Raby Powers, et al. 2005[**57**]**	USA	Elementary school	Qu	Students in second and third grades	
				702	398

*RCT* randomized controlled trial, *Qu* quasi-experimental

**Table 3:** Characteristics of interventions and results of included studies

Author, year		Intervention					Results (increase dairy intake significantly)
	Description (type)	Type of intervention	Only dairy intake OR Mix	Duration (Week)	Frequency—every	Dietary assessment method	
**Kimura, et al. 2013[**33**]**	Participants in the intervention group participated in the Sumida TAKE10! Program. It consisted of a general lecture by a researcher on the importance of dietary variety and 5 educational sessions.	Educational	Mix	12	2 weeks	FFQ	Yes
**Duncanson, et al. 2013[**34**]**	The intervention involved dissemination of the Tummy Rumbles interactive CD and the Raising Children DVD at baseline in September 2009, accompanied by written instructions for optimal use. The Tummy Rumbles interactive nutrition education CD is a self-directed resource that was adapted from an early childhood nutrition education program for child care staff and parents. The resource is divided into modules that include the 5 food groups, dietary fats, fussy eaters, healthy lunchbox ideas, food budgeting, and reading food labels.	Educational	Mix	48	–	Australian Toddler Eating Survey (ATES)—FFQ	No
**McCarthy, et al. 2007[**35**]**	“Fitness” intervention group participants received instruction on skills training in a balanced regular exercise regimen (muscle strengthening, flexibility enhancement and aerobic conditioning), and nutrition education promoting a low-fat, complex carbohydrate-rich diet, emphasizing the cancer-preventive benefits of increased fruit, and vegetable intake.	Educational	Mix	8	Week	The National Cancer Institute (NCI) Health Habits and History Questionnaire (Block Food Frequency instrument–NCI version 02.1)	No
**Casazza, et al. 2006[**36**]**	A computer-based intervention (CBI) program using an interactive, animated CD-ROM aimed at changing in eating behavior and physical activity patterns among high school students. Education programs had an emphasis on healthy lifestyle habits (diet and physical activity).	Educational	Mix	24	2 weeks	24-h recall	Yes
**DeBar, et al. 2006[**37**]**	Behavioral intervention (bimonthly group meetings, quarterly coaching telephone calls, and weekly self-monitoring) designed to improve diet and increase physical activity.	Educational	Mix	96	1 week	24-h recall	Yes
**DeBar, et al. 2009[**38**]**	Participants were urged to consume 1350 mg/day of calcium (four glasses of milk a day or the equivalent) and eight servings of fruits and vegetables, as well as to participate in exercise. Participants were encouraged to use the study website at least once a week for the duration of the 2-year study.	Educational	Mix	96	1 week	24-h recall	Yes
**Dawson, 2006[**39**]**	The intervention, based on Social Cognitive Theory, consisted of five consecutive nutrition education lessons about requirements, food sources, and health benefits of dairy.	Educational	Dairy	5	1 week	The Dairy Self-Efficacy Scale	Yes
**Gates, et al. (1) 2013[**40**]**	School nutrition program including policy, education, food provision, and family and community involvement. An informative handout for parents was given to students (influencing the home environment and role models, and influencing self-efficacy). Healthy breakfast and snacks were provided (including vegetables or fruit, whole grains, protein sources, and milk or milk alternatives).	Multiple	Dairy	5	Day	A 24-h recall and food frequency questionnaire	No
**Gates, et al. (2) 2013[**41**]**	Supplementary milk and alternatives program for snack.	Providing	Dairy	48	1 day	A 24-h recall and food frequency questionnaire	Yes
**Lo, et al. 2008[**42**]**	The intervention was also developed taking into account constructivist theory of learning. The modules aimed to enhance knowledge and understanding about the importance of variety, balanced, and moderation in making wise beverage choices (fruit juice 100%, milk, and water) instead of sugary drinks.	Educational	Dairy	6	Weeks	Beverage frequency questionnaire	No
**Naghashpour, et al. 2014[**43**]**	Nutrition education program based on the Health Belief Model (HBM). A lesson plan of nutrition education was structured.	Educational	Mix	8	Week	Food frequency questionnaire	Yes
**Olson, et al. 2008[**44**]**	Healthy teens utilized a personal digital assistant (PDA)-based screener that provided the clinician with information about a teen’s health risks and motivation to change.	Educational	Mix	24	Week	Study-specific questionnaire	Yes
**O’Connell, 2005[**45**]**	Intervention components included: (1) nutrition education through curriculum, school dinners, and mailing information to families and (2) changes to cafeteria environments to increase the availability and awareness of fruits, vegetables, and dairy products.	Educational	Mix	23	Day	FFQ	No
**Singhal, et al. 2010[**46**]**	Multi-component model of nutrition and lifestyle education. The multi-component model included seven components of nutrition and lifestyle education aimed for changing the knowledge, behavior, and risk profile.	Educational	Mix	24	Day	Study-specific questionnaire	Yes
**Watson, et al. 2009[**47**]**	Students in the intervention group completed 18-week nutrition-related courses. The major topics for nutrition and food science include influences on eating patterns and habits; processes of digestion, absorption, and metabolism of food; major nutrients and their functions; U.S. dietary guidelines; recommended dietary allowances; diet, disease, and weight control; planning nutritious and appealing meals; proper table service and manners; food labels and consumerism; cooking and food preparation terms; measuring and cooking equipment; proper food storage; laboratory experience in preparing foods.	Educational	Mix	18	Day	Study-specific questionnaire	Yes
**Wordell, et al. 2012[**48**]**	Two of six middle schools allowed only bottled water in vending machines, only milk and fruit on à la carte menus, and offered a seasonal fruit and vegetable bar.	Providing	Mix	144	Day	Modified FFQ	Yes
**Yamaoka, et al. 2011[**49**]**	Each participant in the intervention group received twelve sessions of group counseling aimed at increasing energy intake at breakfast by modifying dietary intake and adopting appropriate habits.	Educational	Mix	24	2 weeks	Modified FFQ	Yes
**Finnell, et al. 2017[**50**]**	The 12-week 1% Low-Fat Milk Has Perks! Intervention ran relying on television and radio commercials, print advertisements, billboards and bus wraps, point-of-sale promotional items, and digital media.	Multiple	Dairy	12	Week	Milk sales by type (whole, 2%, 1%, and nonfat milk)	Yes
**Bernstein, et al. 2002[**51**]**	Nutrition education was designed to increase fruit, vegetable, and calcium-rich food consumption. The education program was provided through home visits, phone contacts, and letters.	Educational	Mix	24	2 weeks	FFQ	Yes
**Ni Mhurchu, et al. 2010[**52**]**	Price discounts plus nutrition education. The price discount intervention consisted of an automatic 12.5% price reduction on all eligible healthier food products. Also, participants were mailed a printed package of food group-specific nutrition information by mail.	Multiple	Mix	24	Month	Electronic scanner sales data	Yes
**Foster, et al. 2014[**53**]**	Intervention stores received a 6-month intervention to increase the purchase of recommended healthier items in 5 food and beverage categories. Strategies included (1) multiple facings: increased the number of facings of the recommended products; (2) prime placement: placed recommended products at arm/eye level and in the middle of the category aisle and reordered types of milk so that 2% milk was located on the left-hand side of the dairy case followed by 1%, skim and then whole milk; (3) signage: placed call-out signs with the recommended product’s name and price, and shelf runners below recommended products; and (4) secondary placement: mimicked shelf strategies (1 and 2) in all secondary placements (end caps, dead space stacks, etc.).	Point-of-sale	Mix	48	Week	Supermarket milk sale data	Yes
**Freedman and Nickell, 2010[**54**]**	“Snack Smart” workshops, based on social cognitive theory, were conducted to assess changes in consumption of targeted food items. The program consisted of a 3-week, 6-h series of 5 workshops repeated in 8 different branch libraries over 3 months. Weekly 90-min after-school nutrition workshops were book-ended by two 45-min week-end workshops—the first involving parents and the last engaging parents and children.	Educational	Mix	12	Week	FFQ	No
**Friedman, et al. 2007[**55**]**	The dietary intervention was focused on the family with small group sessions for the parents and for the children separately. There were lessons on the milk group (encouraged skim milk, low-fat cottage cheese, and low-fat puddings), grain group (encouraged adding cereals and whole-grain breads), and meat group (encouraged low-fat meat and how to identify and prepare low-fat meat, and demonstrated convenience meals with low-fat ingredients).	Educational	Mix	350	Week	24-h dietary recalls	Yes
**Hovell, et al. 2009[**56**]**	Children were taught how to engage in eat calcium-rich foods. Parents were taught behavior management techniques to modify children’s behaviors.	Educational	Dairy	8	Week	24-h dietary recalls	Yes
**Raby Powers, et al. 2005[**57**]**	Social cognitive theory-based nutrition education program to increase fruit, vegetable, and calcium-rich food consumption.	Educational	Mix	6	Week	Pizza please, a specially designed interactive evaluation tool	No

Abbreviations: *FFQ* food frequency questionnaire

Participants in four studies were adults and the elderly. Meanwhile, children and adolescents were the point of research in 2 and 17 studies, respectively. Totally, the number of subjects in the intervention and control groups was 6939 and 6753, respectively.

Educational interventions were used in 19 studies. In addition, three studies focused on the sale and providing dairy products and three studies used multiple-level interventions. In six studies, increasing the intake of dairy products was the primary goal and in 19 studies multi-component aims were applied. Regarding the duration of interventions, a maximum of 350 weeks and a minimum of 5 weeks were performed. The intervention was repeated daily in six studies, once a week in 13 studies, once every 2 weeks in 4 studies, and every month in 1 study. In one study, the time of repeating the intervention was not mentioned [34].

### Location/field of study

The majority of studies were performed at school level (*n*: 11), of which six studies were effective in increasing dairy intake. There were 6 studies at the community level, of which 4 were effective. There were 5 studies in health centers, all of which confirmed the effectiveness of the interventions. Fewer interventions were performed in the supermarkets (*n*: 3); the results of all 3 studies showed the effectiveness of the interventions.

### Target group

Adolescent groups were the most common target groups (*n*: 17), with a total of 12 studies confirming the effectiveness of the interventions. Children were the target group in two studies, of which none were effective. The adults and older people were the target group of four studies, three of which were effective. The supermarket intervention was in three studies, all of which were effective in increasing the intake of dairy products.

### Type of intervention

Most of the studies (*n*: 19) were educational, including training with physical exercise, home visits, telephone calls, using the web and technology tools, etc. with approximately 70% of studies succeeding in the increase of dairy consumption when using this type of intervention [33, 36–39, 43, 47, 49, 56]. There were two educational interventions performed among the elderly people, including home visits and telephone follow-ups; both interventions were successful [33, 51]. In the children and adolescents’ group, interventions were training through technology tools [34, 36, 38, 44, 45], peer education in schools [36, 38, 40, 42, 45, 46], pamphlets [43, 45, 46, 54], and training with snacks [40, 48].

Multiple interventions (*n*: 2) also included public advertisement and lowered prices for healthy products in schools or supermarkets; all of these interventions were effective [40, 50].

Providing dairy products for children in schools [41, 48], and increased exposing with the recommended products or prime placement of healthy products in markets were some other types of interventions [53].

### Increasing dairy products consumption as a main goal

In six studies, increasing the intake of dairy products was the primary goal, of which four were effective (66.6%). Moreover, of the 19 studies with other goals, 14 were effective (73.6%) in increasing the intake of dairy products.

### Study duration

The included studies were divided into four groups: studies with less than 8 weeks, including eight studies, of which four were effective; studies with nine to 24 weeks, including four studies, of which two were effective; studies with 24 to 48 weeks, including nine studies, of which eight were effective, and four studies with more than 48 weeks, of which all were effective.

### Intervention frequency

Six studies were repeated daily (repetition due: 1 day), four of which were effective. Most studies (*n*: 13) had a weekly repeat interval, nine of which were effective. Four studies were repeated every 2 weeks, all of which were effective. Also, one study was repeated every 1 month, which was effective.

Interventions reported in 18 studies were effective in increasing dairy consumption and ineffective in seven studies. The effectiveness of the interventions based on the studied variables (country, location/field of study, participants, type of intervention, combination/specific dairy intervention, duration, and frequency) is shown in Table 4. In *low- and middle-income countries* (LMIC) and high-income countries 100% and 70% of the interventions were effective, respectively. Interventions in health centers and supermarkets were more effective than community- and school-level interventions. Interventions in adolescents as target groups were more effective than children, middle-aged, and the elderly people. Also, multiple interventions and changes in buying/selling habits were more effective than educational interventions. Combined interventions to modify dairy intake were more effective than specific interventions. Interventions longer than 24 and 48 weeks were more effective than shorter interventions. Also, interventions repeated every 2 weeks and every month were more effective than interventions repeated daily or weekly.

**Table 4:** The effectiveness of interventions on increasing dairy consumption based on the study variables

Variable	Subgroups	Number of interventions	Number of effective interventions
Country	High-income countries	23	16
	Low- and middle-income countries (LMIC)	2	2
Location/context of study	Community	6	4
	Schools	11	6
	Healthcare centers	5	5
	Supermarkets	3	3
Participants	Children	2	0
	Adolescents	17	12
	Adults and elders	4	3
	Supermarkets	3	3
Type of intervention	Educational	19	13
	Multiple	3	2
	Change in purchase/sell or providing	3	3
Only dairy intake or multicomponent intervention	Combination	19	14
	Only dairy intake	6	4
Study duration	8 weeks and less	8	4
	9 to 24 weeks	4	2
	24 to 48 weeks	9	8
	48 weeks and more	4	4
Intervention frequency	Everyday	6	4
	Every week	13	9
	Every 2 weeks	4	4
	Every month	1	1

### Quality assessment of studies

As can be seen in Tables 5 and 6, all studies had one or more domains characterized as high risk. Also, all studies had good quality in terms of having more low-risk domains than high-risk ones. Ten studies had selection bias due to a non-random selection of participants. Most of the studies had performance bias due to the unblinding of participants and personnel**.**

**Table 5:** Quality assessment of the included RCT studies

Author^*^	Was true randomization used for the assignment of participants to treatment groups?	Was allocation to treatment groups concealed?	Were treatment groups similar at the baseline?	Were participants blind to treatment assignment?	Were those delivering treatment blind to treatment assignments?	Were outcomes assessors blind to treatment assignment?	Were treatment groups treated identically other than the intervention of interest?	Was follow-up complete and if not, were differences between groups in terms of their follow-up adequately analyzed?	Were participants analyzed in the groups to which they were randomized?	Were outcomes measured in the same way for treatment groups?	Were outcomes measured in a reliable way?	Was appropriate statistical analysis used?	Was the trial design appropriate?
Kimura, et al. 2013	Y	Y	Y	N	N	U	Y	Y	N	Y	Y	Y	Y
Duncanson, et al. 2013	Y	Y	Y	Y	U	U	Y	Y	Y	Y	Y	Y	Y
McCarthy, et al. 2007	Y	Y	Y	U	Y	Y	Y	Y	U	Y	Y	Y	Y
Casazza, et al. 2006	Y	Y	Y	U	U	U	Y	Y	Y	Y	Y	Y	Y
DeBar, et al. 2006	Y	Y	Y	N	Y	U	Y	Y	N	Y	Y	Y	Y
DeBar, et al. 2009	Y	Y	Y	U	U	U	Y	Y	N	Y	Y	Y	U
Dawson, 2006	N	U	Y	U	U	U	U	Y	U	Y	Y	Y	Y
Lo et al. 2008	U	U	Y	U	U	U	U	Y	Y	Y	Y	Y	Y
Naghashpour et al. 2014	Y	Y	Y	U	U	U	Y	Y	Y	Y	Y	Y	L
Olson, et al. 2008	N	N	Y	U	U	U	Y	Y	Y	Y	Y	Y	Y
O’Connell. 2005	U	U	Y	U	U	U	Y	Y	Y	Y	Y	Y	Y
Singhal, et al. 2010	U	Y	Y	U	U	U	Y	Y	Y	Y	Y	Y	Y
Yamaoka, et al. 2011	Y	Y	Y	Y	Y	Y	Y	Y	U	Y	Y	Y	Y
Finnell, et al. 2017	U	Y	Y	N	U	U	Y	Y	U	Y	Y	Y	Y
Bernstein, et al. 2002	Y	Y	Y	N	U	U	Y	Y	Y	Y	Y	Y	Y
Ni Mhurchu, et al. 2010	Y	Y	Y	N	U	U	Y	Y	U	Y	Y	Y	Y
Foster, et al. 2014	Y	Y	Y	U	U	U	Y	Y	U	Y	Y	Y	Y
Hovell, et al. 2009	Y	Y	Y	U	U	U	Y	Y	U	Y	Y	Y	Y
Friedman, et al. 2007	U	U	Y	U	U	U	Y	Y	Y	Y	Y	Y	Y
Raby Powers, et al. 2005	U	U	Y	U	U	U	Y	Y	Y	Y	Y	Y	Y

*U* unclear, *N* no, *Y* yes

^*^All the studies are considered as low risk of bias

**Table 6:** Quality assessment of the included quasi-experimental studies

Author^*^	Is it clear in the study what is the “cause” and what is the “effect”	Were the participants included in any comparisons similar	Were the participants included in any comparisons receiving similar treatment/care, other than the exposure or intervention of interest?	Was there a control group?	Were there multiple measurements of the outcome both pre and post the intervention/exposure?	Was follow-up complete and if not, were differences between groups in terms of their follow up adequately described and analyzed?	Were the outcomes of participants included in any comparisons measured in the same way?	Were outcomes measured in a reliable way?	Was appropriate statistical analysis used?
Yeudall, et al. 2005	Y	Y	Y	Y	Y	U	Y	Y	Y
Gates, et al. (1) 2013	Y	Y	Y	N	Y	Y	Y	U	Y
Gates, et al. (2) 2013	Y	Y	Y	N	Y	Y	Y	Y	U
Watson, et al. 2009	Y	Y	Y	Y	Y	Y	Y	Y	Y
Wordell, et al. 2012	Y	Y	Y	Y	N	Y	Y	Y	Y
Freedman and Nickell 2010	Y	Y	Y	N	Y	Y	Y	Y	Y

*U* unclear, *N* no, *Y* yes

^*^All the studies are considered as low risk of bias

## Discussion

Out of 521 retrieved articles, 25 were finally included in the study. The reported interventions were effective in increasing dairy consumption in 18 studies and ineffective in seven studies. As noted above, most interventions were conducted in high-income countries. This could be related to the greater research budgets. In high-income countries, different intervention methods and techniques had been used compared to low- and middle-income ones. The existing differences between these two groups of countries should be considered in applying the intervention types [58–60].

The results showed that the interventions performed in the healthcare centers and supermarkets were more effective than the community- and school-level interventions. In addition, interventions in health centers are more acceptable and better adhered because of the psychological impact of these centers on participants. People who visit these centers tend to have a more positive attitude towards the role of the health system and they usually trust the recommendations of the health system [61–63]. Also, in community-based interventions, participants are usually adults and the elderly and changing food habits and behaviors in these groups is a really challenging task compared to children and adolescents. Therefore, it seems that designing and implementing interventions to increase dairy consumption can be better achieved if combined with the collaboration of the health system and the healthcare staff.

Regarding the effectiveness of interventions in supermarkets, all interventions in these centers were control/change interventions in the purchase of dairy products, which were much more effective. These interventions are usually made by offering discounts on dairy purchases (especially low-fat dairy) that appear to be a stronger incentive. It should be noted that a number of purchases were measured in these studies and the amount of dairy consumption was not assessed.

Based on the results, interventions on the children, the middle-aged, and the elderly people were less effective. The two studies performed in children applied multiple methods like policy changing, education, family and community involvement, and educational methods using educational CDs for children and parents. The duration of studies varied from 5 to 48 weeks. The results of review studies in different fields also indicate that the effectiveness of educational interventions on children is low [64–68]. The results of a systematic review and meta-analysis showed that interventions in adolescents were more effective in comparison with children [69]. Reasons for the lower effectiveness of interventions on children may be due to the low dose of the intervention [40], self-administered questionnaires which could lead children to under- or overestimate their intake [42], and lack of out-of-school nutrition programs [54].

Birch and colleagues have also stated that in order to improve primary school children’s healthy food preferences, experiences and strategies are needed to increase availability and accessibility to increase exposure to those foods that will then affect their willingness to taste [70]. The low effectiveness of interventions in middle-aged and elderly people may also be attributed to the consolidation of dietary habits and other behaviors in these people; so modifying their behavior is very difficult [71, 72].

The results of our study showed that most of the interventions were educational, which had lower efficiency compared with interventions such as multiple interventions and changing the purchase patterns. The results of several systematic reviews support this finding [73, 74]. Although many experts believe that the effectiveness of educational interventions alone is in doubt, it seems that training can be effective if it is targeted and accompanied by other interventions to change behavior [75–77]. The findings of this study showed that providing dairy products is one of the effective methods to increase dairy intake. Wordell et al. demonstrated that healthful modifications in the school food environment are associated with positive food behaviors in early adolescents, but there was a cost associated with those changes. So, it seems that, if possible, providing dairy is a suitable way to increase the consumption of dairy products.

Interventions that lasted more than 24 weeks and repeated every 2 weeks and each month were more effective. The likely reason for the effectiveness of long-term interventions may be due to the effect of these interventions on the change of behavior and its stabilization. Because any short-term changes in human behavioral patterns can simply return to the basic state, but the longer-term change increases the likelihood of a sustained change in behavior [78, 79]. Possible reasons for the less effectiveness of interventions that were repeated less frequently (daily and weekly) might be that participants become bored. In addition, most of the interventions that were repeated less frequently were educational and multiple interventions, that were less effective than the other interventions.

The main advantage of our systematic review is the low risk of subjective data selection. Study searches, assessment, and data synthesis were based on predefined criteria and were performed using well-established tools by two independent reviewers. Nevertheless, our analysis had some limitations. First, publication bias cannot be excluded, i.e., ineffective interventions are less likely to be published. Potential limitations existing in the included studies are as follows: unclear or inadequate allocation concealment, no intention-to-treat analysis, inadequate information about controlling confounders, and applying different questionnaires for evaluating dairy intake. One of the most important limitations of the present study is the dispersion and variability of the reported indices for the effectiveness of interventions in the studies. Hence, performing a quantitative analysis (meta-analysis) was impossible.

## Conclusion

According to the results of the present systematic review, three policy options including educational interventions, multiple interventions, and changing the purchase patterns are suggested. It seems that applying all of the interventions together can be more effective. Interventions in health centers and supermarkets are more effective than the community interventions. However, it should be noted that the implementation of the proposed interventions and settings depends on the limitations, resources, and facilities of different countries. So, long-term and high-frequency interventions focusing on increasing dairy products intake are suggested in different settings and countries.

## Data Availability

If requested, data will be available at https://hsri-en.tbzmed.ac.ir/.
